# Endovascular repair for acute mesenteric ischemia: case report

**DOI:** 10.1186/1471-2318-11-S1-A17

**Published:** 2011-08-24

**Authors:** L Fiengo, C Paciotti, G Patrizi, L Venturini, A Pucci, F Fanelli, A Bruni, M Allegritti, A Redler

**Affiliations:** 1 Department of Vascular and General Surgery, Faculty of Medicine “La Sapienza”, Roma, Italy; 2 Department of Vascular and Interventional Radiology, Faculty of Medecine “La Sapienza”, Roma, Italy

## Background

Acute mesenteric ischemia (AMI) is an abdominal emergency caused by: embolism (40-50%), with Superior Mesenteric Artery (SMA) thrombosis (20-25%), mesenteric venous thrombosis (5%) and non occlusive mesenteric ischemia (20%). The mortality rate is high and ranges from 64 to 93%.

## Methods

We present a case of a 75-year-old patient with acute occlusive mesenteric ischemia that was successfully treated with endovascular intervention.

Angiography revealed high-grade stenosis of the proximal tract of the SMA. Immediate option for endovascular therapy was made, and a MARIS self-expandable 6x40 mm stent was positioned. The patient was discharged 2 days after with full recovery from the symptoms.

## Results

Control angiography showed the correct apposition of the stent and regular flow inside. The patient was symptom free 12 months later.

## Conclusions

We suggest that the endovascular technique is a valid option in patients with AMI preventing intestinal infarction.

**Figure 1:**
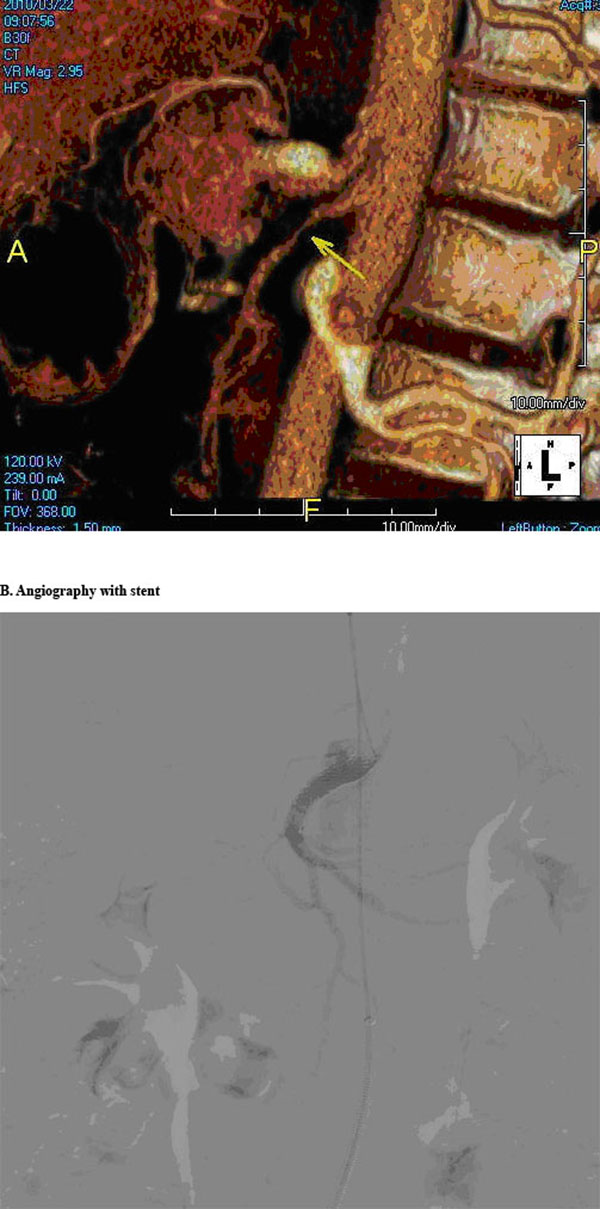
A) Preoperative angiography. B) Angiography with stent apposition
